# Theranostic Implications of Nanotechnology in Multiple Sclerosis: A Future Perspective

**DOI:** 10.1155/2012/160830

**Published:** 2012-12-30

**Authors:** Ajay Vikram Singh, Manish Khare, W. N. Gade, Paolo Zamboni

**Affiliations:** ^1^Department of Biotechnology, University of Pune, Ganeshkhind Road, Pune 411 007, India; ^2^Center for Biotechnology and Interdisciplinary Studies, Rensselaer Polytechnic Institute, Room 2145, 110 8th Street, Troy, NY 12180, USA; ^3^Department of Applied Sciences, Maharashtra Academy of Engineering, Alandi (D), Pune 412 105, India; ^4^Centre for Vascular Disease, University of Ferrara, 41100 Ferrara, Italy

## Abstract

Multiple Sclerosis is a multifactorial disease with several pathogenic mechanisms and pathways. Successful MS management and medical care requires early accurate diagnosis along with specific treatment protocols based upon multifunctional nanotechnology approach. This paper highlights advances in nanotechnology that have enabled the clinician to target the brain and CNS in patient with multiple sclerosis with nanoparticles having therapeutic and imaging components. The multipartite theranostic (thera(py) + (diag)nostics) approach puts forth strong implications for medical care and cure in MS. The current nanotheranostics utilize tamed drug vehicles and contain cargo, targeting ligands, and imaging labels for delivery to specific tissues, cells, or subcellular components. A brief overview of nonsurgical nanorepair advances as future perspective is also described. Considering the potential inflammatory triggers in MS pathogenesis, a multifunctional nanotechnology approach will be needed for the prognosis.

## 1. Introduction

**Figure 1:**
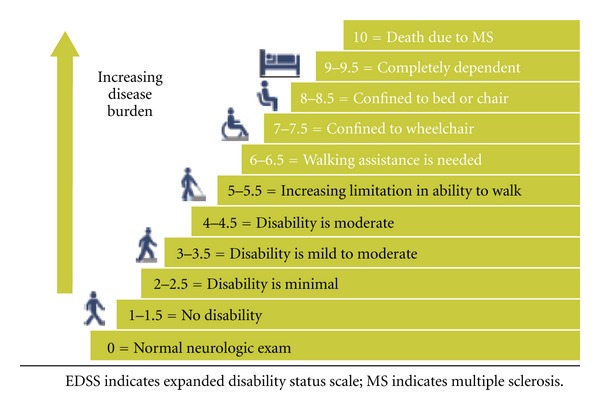
*Scaling the progression of disability*. EDSS score in MS (published with permission of [11]).

Multiple sclerosis (abbreviated MS), disseminated sclerosis, and encephalomyelitis disseminata are synonyms to an autoimmune condition rather than disease in which cells from immune lineage attack nervous system bringing demyelination [1, 2]. It has passed more than a century since Charcot, Carswell, Cruveilhier, and others described the clinical and pathological characteristics of multiple sclerosis [3]. The onset of this enigmatic and progressive disorder of white matter of central nervous system (CNS) occurs in young age and is more common in females [4]. There are clinically defined MS patients with a prevalence that ranges between 2 and 150 per 100,000 [5]. High resolution magnetic resonance imaging (MRI) and spectroscopic analysis are fundamental tools for clinicians, assisting in prognosis of the disease [6]. This further helps in monitoring pathological progression and course of treatment of the disease. MS affects the ability of nerve cells in the brain and spinal cord to communicate with each other. Neurons communicate with each other by sending electrochemical signals called action potential along extending processes called axons, which are wrapped in an insulating lipoprotein, a dielectric substance called myelin [7, 8]. In MS, body's own immune system attacks and damages the myelin. In abbreviated MS, the terms “scleroses” refers to scars, plaques, or lesions, those appear as “multiple” patches along white matter of cerebrospinal regions of the brain and spinal cord that can be seen in drawing from a Carswell book in 1838, the first clinical picture so far drawn [7, 8]. Many studies have shown the mechanism involved in disease process, but causes are unknown though indicate multifactorial theories including genetics and immune infections as the central role [9]. Neurological symptoms with physical and cognitive disability; new symptoms occurring either in discrete attacks (relapsing forms) or slowly accumulating over time (progressive forms) are delineated factors to clinically assess the disease, but permanent neurological problems aggravate as the disease advances [10]. The Kurtzke Expanded Disability Status Scale (EDSS) measures the progression of MS using a rating scale between 0 and 10 which defines disability status with progression of the diseases (Figure 1) [11]. The present articles give an overview of how nanotechnological implications can be utilized to improve quality of life (QoL) in MS patients which has been unrealized so far. We principally focus on theranostic approaches at nanoscale which has been successfully implemented in other CNS disorders (AD, PD, ALS, etc.) and open promising avenues in MS too [12, 13]. 

In this short perspective, we will first discuss nanotechnology-based therapeutic approaches utilizing nanoparticles as programmed drug delivery vehicle for neuroprotections and neuronal enhancement in diseased brain. Our discussion will be focused mainly on material design strategies circumventing to cross blood barrier and neurovasculature. Then, we represent an overview of advances made in nanodiagnostic assessment using NPs as contrast agent and/or multipartite system to deliver therapeutic + diagnostic (theranostic) together.

## 2. Nanoscience and Technology in CNS Disorders

In recent decades, nanotechnology has emerged as an impressive tool of treating neurological disease, with the radical changing the way we approach the CNS-targeted neurotherapeutics in the past. This lead to promising progress into treatments for diseases of the brain and CNS in spite of limited therapeutic options for many patients with neuropathology worldwide [14]. The major advantage of nanoscale technology supporting therapeutic application in neuropathology stems from nanoengineering and conjugation opportunities of therapeutic molecules with nanoparticles [15].

This in turn supports stability of drug molecule and helps to cross the blood-brain barrier for targeting specific cell signaling in brain. Particularly in nervous system where cells frequently loose the regenerative capacity following *in vivo* injury, nanomolecules are used as matrix to promote neural elongation and support cell survival in damaged cells or act as vehicles for gene delivery to tame molecular responses to endogenous pathological stimuli [16]. A wide variety of nanodevices and nanomaterials with capability to engineer the structure-function relationship matching with nanoscale molecular hierarchy in neuronal system make the nanotechnology powerful tool in treating the neurological disorders [17, 18].

## 3. Blood-Brain Barrier (BBB) and Reticuloendothelial System (RES) in MS

**Figure 2:**
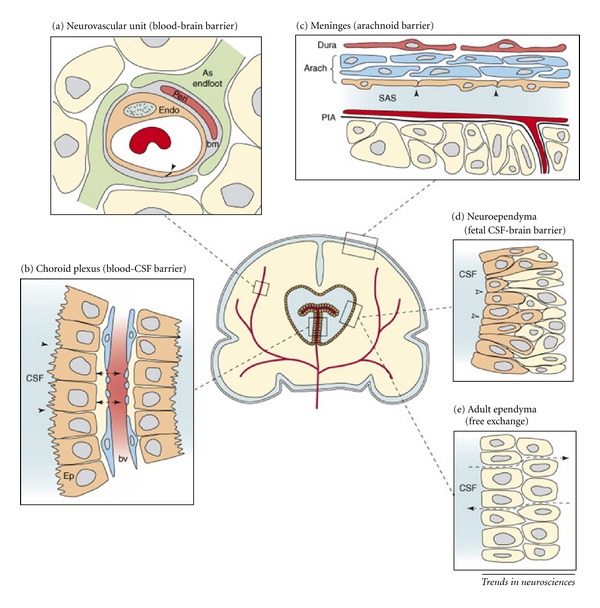
*Barrier interfaces in brain.* (a) endothelial cells (endo) in the neurovascular unit have luminal tight junctions (shown by the arrow) that form the physical barrier of the interendothelial cleft. Outside the endothelial cell is a basement membrane (bm) which also surrounds the pericytes (Peri). Around all of these structures are the astrocytic end-feet processes from nearby astrocytes. (b) The endothelial cells of choroid plexus blood vessels are fenestrated and form a nonrestrictive barrier (shown by dashed arrows) between the cerebrospinal fluid (CSF) and blood vessel (bv). The epithelial cells (ep) have apical tight junctions (shown by arrows) that restrict intercellular passage of molecules. (c) In the meninges, the blood vessels of the dura are fenestrated and provide little barrier function (not shown). However, the outer cells of the arachnoid membrane (Arach) have tight junctions (shown by arrows), and this cell layer forms the physical barrier between the CSF-filled subarachnoid space (SAS) and overlying structures. The blood vessels between the arachnoid membrane and the pial surface (PiA) have tight junctions (not shown). (d) In early development, the neuroependymal cells are connected to each other by strap junctions (shown by arrows) that are believed to form the physical barrier restricting the passage of larger molecules, such as proteins, but not smaller molecules, such as sucrose. (e) The mature adult ventricular ependyma does not restrict the exchange of molecules (shown by dotted arrows). The neurovascular unit (a), blood-CSF barrier (b), and arachnoid barrier (c) are common between developing and adult brain, whereas fetal neuroependyma (d) differs from adult ependyma (e) (cited from [19] with permission from cell press).

The brain barrier occurs along all capillaries and consists of tight junctions around the capillaries that do not exist in normal circulation at the base of the brain [16]. Endothelial cells restrict the diffusion of microscopic objects (e.g., bacteria) and large or hydrophilic molecules into the cerebrospinal fluid (CSF), while allowing the diffusion of small hydrophobic molecules (O_2_, CO_2_, hormones) [19]. Cells of the barrier actively transport metabolic products such as glucose across the barrier with specific proteins (Figure 2). This barrier also includes a thick basement membrane and astrocytic end feet. The strong association of BBB in MS has been widely elucidated with histopathological and molecular changes [20, 21]. The relapsing and progressive episodes in MS “attack” has shown the broken blood-brain barrier in a section of the brain or spinal cord, allowing T lymphocytes to cross over and attack the myelin which gradually leads to complete demyelination [20, 22]. Our recent hypothesis also corroborates that a complex pattern of extracranial venous stenosis determines flow abnormalities such as reflux and blockages in the main extracranial outflow routes, namely, the internal jugulars and the azygos vein [23–25].

This creates a collateralization of the venous outflow with increased mean transit time and reduced perfusion of the brain parenchyma of MS patients [26]. Reduced perfusion is a typical aspect of MS and cannot be explained of course by autoimmunity [27]. It might explain aspects of hypoxia-like conditions in the MS plaques, early axon damage in absence of T-cells and oxidative stress with mitochondria impairment [28]. Another constant feature linked with hampered venous outflow is represented by blood-brain barrier breakdown. The latter may favor erythrocytes diapedesis with iron deposition into the brain parenchyma, which triggers a further local inflammatory response and amplifies the oxidative stress [29]. It will not be an exaggeration if, rather than being a disease of the immune system, MS is termed as a disease of the blood-brain barrier and RES [30]. This is followed by increased intravenous pressure, blood-brain barrier breakdown, and iron deposition into the brain parenchyma, which triggers a local inflammatory response [30]. It will not be an exaggeration if, rather than being a disease of the immune system, MS is termed as a disease of the blood-brain barrier and RES [31].

## 4. Drug Delivery System Crossing BBB

Conjugation of therapeutic peptides or antibodies to the surface of magnetic nanomaterials helped in direct targeting and potential disruption of active signaling pathways of the tumor cell surface [32]. This field further opens potential avenues of the magnetic nanoparticles in translation studies in the brain pathology, such as imaging and targeting the sclerotic lesion with growth factor to treat the lesions in MS patients [33]. Delivery of conventional therapeutic to brain and CNS disorder represents a formidable challenge due to the presence of the blood-brain barrier and complex interplay of endothelial cells, astrocyte and pericytes (RES) at BBB in the normal brain [34]. The active targeting strategy with site-specific ligands binding increases penetration and surface nanoengineering of NPs, which provided new ways to control pharmacokinetics and bioavailability of CNS-related drugs across BBB and RES [18, 35]. PEGylation of liposome and maintaining the particle diameter at <100 nm help in combating problem associated with conventional liposomes (aggregation, short half-lives, modest transport capacity across the blood-brain barrier, and rapid RES clearance) by receptor or absorptive-mediated transcytosis [36]. Coating the liposome surface with monoclonal antibodies to glial fibrillary acidic proteins, transferrin receptors or human insulin receptors (nanoliposome) further help in escaping RES and BBB and delivering therapeutic genes [37, 38].

## 5. Polymeric Artificial Cells

Nanotechnology allows precise control over *in vitro* mimesis of molecular features at nanoscale for controlling material-cell interactions. This in turn induces specific developmental processes and cellular responses including differentiation, migration, and outgrowth in neuronal cells [39, 40]. Inspired by polymeric artificial cells [41], hollow fibres or three-dimensional polymeric structures as a capsule, protected from immune rejection by an artificial semipermeable membrane, have been made by macro- and microencapsulation [13, 42, 43]. The cell-loaded capsules can be implanted into the damaged brain area favoring the local, targeted, and long-term release of drugs or proteins [42, 44, 45]. The microcapsule loaded with ciliary neurotrophic factor- (CNTF-) producing fibroblasts encapsulated into polymers with a vitrogen matrix and implanted intrathecally in clinical trial of amyotrophic lateral sclerosis in mouse (an animal model of MS) demonstrated in situ sustained delivery of CNTF without any immune- or cytotoxicity [13, 46]. However, results obtained in this trial are matter of further investigation to determine whether enhanced survival is secondary to the transplant environment and/or the epithelial cells fibroblasts [47].

## 6. Carbon Base Nanomaterial

Carbon nanotubes (CNTs) have electrical, mechanical, and chemical properties, and nanoscale features of CNTs make them better suited as an interface with neurons for stimulating and recording neural activity [48, 49]. Notably, purified carbon nanotubes used as substrate/scaffolds reported to promote the formation of nanotube-neuron hybrid networks, able per se to trigger neuron integrative abilities, network connectivity, and synaptic plasticity [50, 51]. The stable interaction of carbon nanotube platforms with stem cell lineage sparked its versatile application in nerve tissue engineering to probe and augment cell behavior [50, 52]. It further opens new routes to treat CNS in MS pathology for nongenetic manipulations of neuronal performance and network signaling *in vivo* as demonstrated for contemporary disorders [18, 53].

## 7. Polymeric Micelles and Nanoparticles

Recent advances in nanoparticle design have demonstrated tremendous potential in engineering matrix chemistry of nanoparticles to design stimuli responsive polymeric nanocarriers [54].

**Figure 3:**
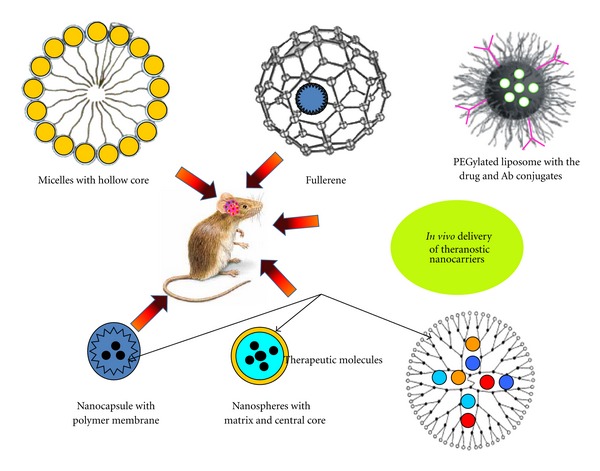
Polymeric nanocarriers in current CNS disorders. Micelle core is promising site for loading insoluble therapeutic agents, while liposome can be targeted by conjugating Ab linker. Fullerene cage surfaces can be functionalized for targeted delivery. Branched dendrimers and nanocapsules are potential nanotheranostic agents.

Versatile strategies and protocols provide platform to tune intracellular stimulus (e.g., reducing nature of the cytosol compared with the extracellular space or the endosomal pH drop) [55] or to an external stimulus (e.g., applied magnetic field or exposure to a specific wavelength of light) [56, 57]. The specific stimulus helps in triggering the drug release in situ via covalent bond cleavage between carrier (vehicle) and cargo (drug) at target (e.g., cell or tissue) [58, 59]. The researches have designed “drug depots” with controlled release micelle-drug compositions. The core-shell architecture of amphiphilic block copolymers and micelle makes them particularly attractive for drug delivery vehicle [60]. The core can incorporate considerable amounts (up to 20%–30% weight) of water-insoluble drugs (Figure 3). Polymeric shell increases their pharmacokinetic release by preventing nonspecific interactions with enzymes, serum proteins, and nontarget cells. This further inhibits premature degradation and release of drug in dispersions, and the drug is released from the micelle via diffusion at specified target [13, 58]. Inspired by fenestration in tumor, targeting brain tumor vasculature with circulating nanoparticles with inherent accessibility of vascular components during angiogenesis opens many perspectives in MS patients [61]. It involves complex interplay of upregulation and secretion of growth factors, which activates endothelial cells to secrete matrix metalloproteinase (MMPs) [62], which degrade the extracellular matrix (ECM) near the brain lesions [63]. This actively provides access to other cells to migrate at lesion site promoting ECM remodeling and cell proliferation [64, 65]. Liposome containing therapeutic has been targeted to vasculature by the attachment of the arginine-glycine-aspartic acid (RGD) peptide and demonstrated many marked fold improvement in drug efficacy compared with the free drug [66, 67].

## 8. Emerging Concept of Nanoneuroprotection

The aim of neuroprotection is to limit neuronal dysfunction/death after chronic CNS injury as happens in AD, PD, and MS, which results in salvage, recovery, or regeneration of the nervous system [68, 69]. Many nanomaterials with antioxidant properties have shown the potential to eliminate reactive oxygen species (ROS) in the brain. Particularly, cerium and yttrium oxides (CeO_2_ and Y_2_O_3_) NPs showed ROS mitigation in *in vitro* conditions using hippocampal neuronal cells [13, 70]. Another class of novel nanomaterial receiving attention for neuroprotection is fullerene and its derivatives. The three-dimensional arrays of evenly spaced carbon atoms with high degree of unsaturated bonds display high reactivity with oxygen free radicals and possess antioxidant and free radical scavenger properties as water-soluble derivatives [12, 71]. Glutamate receptors, which mitigate neuronal toxicity via intracellular calcium influx and limiting excitotoxicity, were shown by fullerenol [polyhydroxylated C60] and carboxyfullerene [malonic acid C60 derivative], respectively, in an *in vivo* mouse model of familial amyotrophic lateral sclerosis, an animal model for MS [72, 73]. Here, fullerenols might partly inhibit glutamate receptors, as they had no effect on GABAA or taurine and/or lowered glutamate-induced elevations in intracellular calcium, which is an important mechanism of neuronal excitotoxicity involving receptors [74]. This opens a wide perspective and scope of fullerene derivatives in MS therapeutics and diagnosis (theranostics); nonetheless, lipid peroxidation, as well as decrease of glutathione in the gill cells, is a major concern considering it for neuroprotecting agent [68]. Compromising the toxicity of fullerenes with its potential theranostic applications in biomedicine, researchers found ways to modify CNTs and fullerene surfaces as demonstrated using single-walled CNTs (SWCNTs) and multiwalled (MWCNTs) via purification and chemical modification, aimed to increase solubility and decrease toxicity [75, 76]. 

Neuroinflammation is another hallmark of MS, and evidence from animal models suggests a mutual interplay of microglia, astrocyte, and T cells, other than demyelination [77]. In advance stages of MS, targeting neuroinflammation as potent therapeutic strategy has shown a promising strategy [78, 79]. However, drug diffusion across BBB and neurovasculature of MS brain yet remain a major challenge. In a recent approach based on nanomaterials (polyamidoamine dendrimers), authors demonstrated targeted delivery of therapeutic localized in activated microglia and astrocytes of diseased brain which suppresses neuroinflammation and leads to a marked improvement in motor function [15, 80]. The nanomaterial-based protocol in the current work provides an opportunity for clinical translation and opens window of opportunity for the treatment in advance MS pathology as successfully shown for animal models of MS [78, 81]. 

## 9. Implications of Nanotechnology in Neurosurgery: Nanobodies Reaching MS Lesion Sites

**Figure 4:**
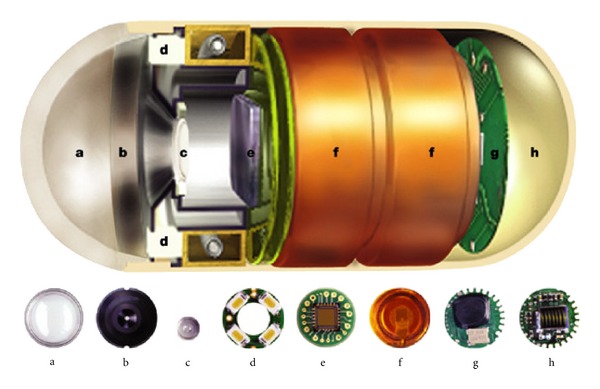
*The M2A capsule camera.* The device consists of a disposable plastic capsule that weighs 3.7 grams and measures 11 mm in diameter by 26 mm in length. The contents include an optical dome (a), a lens holder (b), a short focal-length lens (c), six white-light-emitting diode illumination sources (d), complementary metal oxide silicon (CMOS) chip camera (e), two silver oxide batteries (f), a UHF band radio telemetry transmitter (g), and an antenna (h) (published with permission from [96]).

In the last decade, nanoscience and technology (NST) evolved as applied field beyond notion and speculations and spurred a strong impact not only in clinical sciences but also in almost all occupations of human reach [82–84]. Particularly in neurobiology and clinical surgery, until recently, it proved to be sophisticated technique to enable micronanoscale cellular engineering and manipulations [85]. The nanosurgery will be the next medical frontier in neurobiology, which will eventually make substantial contributions to the advancement of neurosurgery in the near future [86, 87]. Nanosurgery will involve nanoimaging and clearing of the defects/disorders at cellular and subcellular levels. Synthetic nanoscale magnetic materials (e.g., cytobots and karyobots) with impressive properties will promote regeneration in damaged axon and halt deleterious processes (e.g., hemorrhaging) via nanomanipulations [88, 89]. Nonsurgical nanorepairs and nanoneuromodulations will enable monitoring or stimulating diseased neurons. This will involve interaction of the nanobodies with lesions in nervous system in deep MS brain through electrical and/or electrochemical (e.g., neurotransmitter; AcH) function by extending neuronal synaptic connections [90]. In the last decade, single-cell nanosurgery was thought to be neurophilosophy, but current tools such as QDs for nanoimaging, femtosecond pulses of near-infrared laser as surgical tweezers, multipartite NPs for neuromodulations, and AFM cantilever for nanomanipulations make it reality [91]. In one approach, researchers inserted AFM tip as sharp needle “nanoscissors” into the cell wall, which was indented by only one micrometer. This was much more delicate than routine clumsy method which is inevitably difficult to control in microcapillary procedures. AFM tip can be successfully inserted into the nucleus to and forth, and cell membrane quickly returns to its original shape [92]. This opens many perspectives for single-cell neurorepair such as coating tip with specific monoclonal antibodies (MAb) to interact with the intracellular protein traffic and enables monitoring the real-time intracellular chemistry. The ability to manipulate optically subcellular structures at submicrometer while minimizing photodamage has strong implications in MS brain lesions where neuronal cell contain a heterogeneous population of healthy and defective organelles. Improving the tools for single-cell and chromosomal nanosurgery and translating it into the cutting edge *in vivo* surgical device will revolutionize the field [93]. The concept of microrobotics and nanobodies with tiny magnetically driven spinning screws intended to swim along veins and carry drugs to infected tissues or even to burrow into diseased brain cells has risen from fiction to facts [93, 94]. In this series, the first Food and Drug Administration (FDA) approved, wirelessly controlled implantable microchip for osteoporosis drug delivery (e.g., human parathyroid hormone fragment (1–34) [hPTH(1–34)]) release device, is in market [95]. Also, the “camera in a pill” is one recent development which enabled surgeons to monitor real-time pathology and drug release in different accessible areas of gastrointestinal tracts (GITs) [96]. Research is underway to develop capsules with noninvasive propulsive and therapeutic capabilities in other realms of medicine to measure pH, temperature, blood perfusion, and intestinal motility during its journey through the systemic circulation (Figure 4) [97].

## 10. Diagnostic Implication of Nanomaterials for *In Vitro* and *In Vivo* Imaging (QDs, SPIONS, Gd)

**Figure 5:**
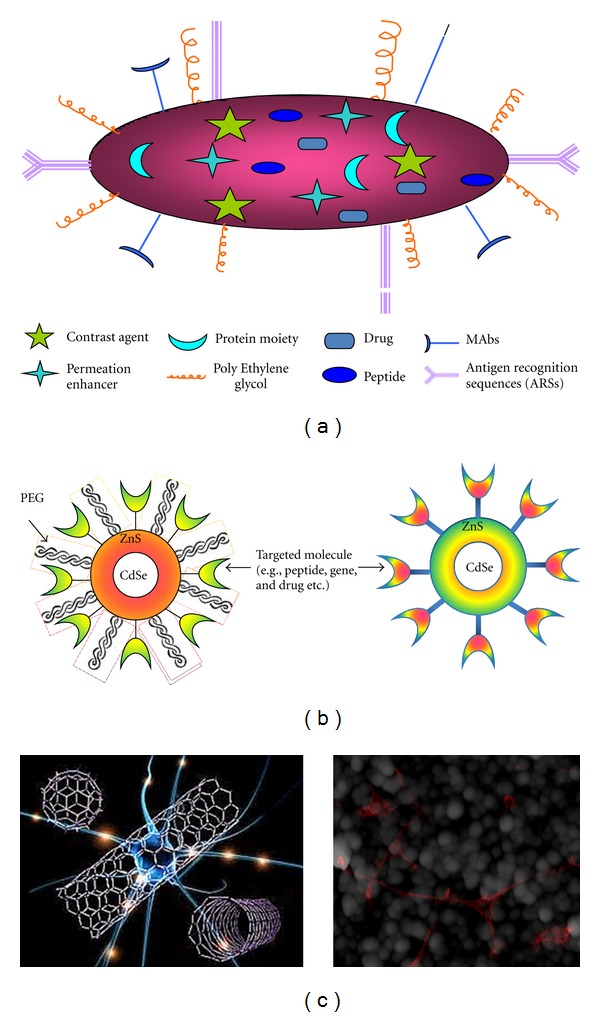
*Multivariate nanocarriers.* (a) multipartite nanostructure with targeting agent, permeation enhancer for BBB and RES, and multicomponent theranostic agent. PEGylation strategy for QDs for increasing uptake and combating BBB (b). (c) demonstrates interaction of neurons with the CNTs and fluorescently labelled neurons interaction with nanostructured surfaces.

DNA-carrier gold nanoparticles- (AuNPs-) based biobarcode assay is competent in amplifying and detecting weak molecular signals up to attomolar concentration @sensitive protein biomarkers in CSF of diseased brain [98]. The bio-barcode assay is capable of amplifying and quantification of connecting molecular loop at ultralow concentration for signal detection, transduction (recording), and signal documentation at molecular scale [99]. These have strong implications in MS, since radio diagnoses (MRI, SWIP, Eco-doppler) are only available gold techniques for early detection of MS brain pathologies [100]. Another ultrasensitive and inexpensive optical method is localized surface plasmon resonance (LSPR) based upon anisotropic silver nanoparticles (AgNPs) nanosensor. The method relies on detecting perturbations in refractive index of the surrounding magnetic field which is an outcome of AgNPs-protein marker interactions at ultralow concentrations [101]. Atomic force microscopy has proven to be a useful technique in understanding molecular interactions at bionanointerfaces [102]. In a recent approach based on a scanning tunneling microscope (STM), authors demonstrated a sandwich-type immune binding assay with cantilever, which is sensitive to tip-to-biosurface interval. The signal transformation analyses give strong indication in the pulse-like peaks of tunneling current, and surrounding concentrations as low as 10 fg/mL can be detected [103]. A fast, ultra-sensitive, and specific nanosensor has been recently devised utilizing two-photon Rayleigh scattering signal emerging from bioconjugated tau protein AuNPs. The method claims to probe as low as 1 pg/mL within half an hour [104]. Furthermore, the implications multipartite NPs and QDs with multiple functionalities to treat ailed neurons have given hope for the future course of MS early diagnosis and cure (Figure 5).

## 11. New Contrast Agent for MRI to Detect Inflammatory Cellular Infiltration in MS

Other than routinely used contrast imaging MRI, new and improved tools to image the cellular and metabolic features of MS are emerging rapidly. Gadolinium-DTPA (Gd-DTPA) is routinely used inflammatory marker in MS [105]. Recently, researchers demonstrated that ultrasmall superparamagnetic particles of iron oxide (USPIO) can visualize cellular infiltration and pluriformity of inflammation in MS more accurately compared to traditional techniques [106]. Interestingly, patterns of USPIO enhancement which have been observed contrary to routine Gd-DTPA exhibiting sensitivity and specificity of MRI in multiple sclerosis can be improved using USPIO (i) focal enhancement, (ii) ring-like enhancement, and (iii) return to isointensity of a previously hypointense lesion [107]. In rat model of ALS, lately, MRI has been utilized to follow labeled T cells with ultrasmall paramagnetic iron oxide (USPIO) NPs *ex vivo* and exhibited an infiltration of CD4+ lymphocyte in the midbrain/interbrain, while CD8+ cells were more confined to the brainstem region [108]. In another work, Machtoub et al. had successfully shown molecular imaging of brain lipid environment of lymphocytes in ALS mouse model using MRI and SECARS microscopy. They were able to detect the pathological regions in ALS rat brain via intravenously injected USPIO NP conjugated with anti-CD4 antibodies [109].

Micro- and nanoparticle technology in particular boosted MRI imaging as contrast agent tremendously [110]. It provide a novel opportunity of incorporating multiple functionalities into a single delivery vehicle, and reports show that, when combined with photoacoustic tomography (PAT), MRI sensitivity increases to picomolar concentration [111].

## 12. Concluding Remark

Albeit applications of nanotechnologies in neurological disorder treatment are in infancy; the potential of using these nanomaterials for treatment and diagnosis of multiple sclerosis opens many promises. Early MS therapy and diagnosis with traditionally means met with limited success outside of a few oral pharmacological agents capable of modifying anti-immune symptoms (e.g., Natalizumab) or physical exercise ameliorating motor dysfunctions. However, early diagnosis of MS would be the best approach in order to prevent irreversible and uncontrollable disability consequence. Many molecular markers and radiodiagnosis approaches successfully demonstrate the disease progress, but sensitivity to recognize onset of the stages achieved recently bases upon nanomaterials contrasts agents base. The nanomaterials nanodiagnostic tools utilize different nanoparticles/nanostructures and are based on different physicochemical interactions that may be utilized either *in vitro *or *in vivo. *Nevertheless, there are still many challenges regarding the immuno/geno/cytotoxicity of nanoparticles and micronanodevices especially in a complex biological milieu like brain with complex network of neuronal cells. 

Yet, a long and puzzling path is ahead to make the envisioned nanoneurosurgical approaches of curing MS diseases as a practical technology and, eventually, a routine clinical practice. The advances in biological microelectromechanical system (Bio-MEMS) and nanoelectromechanical system (NEMS) need to be explored as surgical tool to target and cross the BBB/RES to reach MS brain and perform requisite surgery. Moreover, with inherent complexity of the brain itself and the myriad cellular biochemical responses associated with injury and repair, we need to develop a highly interdisciplinary approach at the biotic/abiotic interface for recognition of disorder, neuroprotection, and neurorepair at the onset of MS. With the significant increases in the prevalence and incidence of MS and related disorders worldwide, the new approaches and interdisciplinary advances are very much needed to fight against the debilitating disorder.
